# Distribution of Acute and Chronic Kidney Disease Across Clinical Phenotypes for Sepsis

**DOI:** 10.1016/j.chest.2024.03.006

**Published:** 2024-03-08

**Authors:** Luca Molinari, Gaspar Del Rio-Pertuz, Priyanka Priyanka, Ali Smith, Joseph C. Maggiore, Jason Kennedy, Hernando Gomez, Christopher W. Seymour, John A. Kellum

**Affiliations:** a Department of Critical Care Medicine, University of Pittsburgh, Pittsburgh, PA; b Center for Critical Care Nephrology, University of Pittsburgh, Pittsburgh, PA; c Department of Developmental Biology, University of Pittsburgh, Pittsburgh, PA; d Division of Cardiology, Department of Medicine, University of Minnesota, Minneapolis, MN; e Department of Translational Medicine, Università degli Studi del Piemonte Orientale, Novara, Italy

**Keywords:** acute kidney disease, acute kidney injury, biomarkers, chronic kidney disease, endotypes, insulin-like growth factor binding protein 7, phenotypes, sepsis, septic shock, tissue inhibitor of metalloproteinases-2

## Abstract

**Background:**

Sepsis is the most common cause of acute kidney injury (AKI) in critically ill patients. Four phenotypes (α, β, γ, δ) for sepsis, which have different outcomes and responses to treatment, were described using routine clinical data in the electronic health record.

**Research Question:**

Do the frequencies of AKI, acute kidney disease (AKD), chronic kidney disease (CKD), and AKI on CKD differ by sepsis phenotype?

**Study Design and Methods:**

This was a secondary analysis of a randomized clinical trial of early resuscitation, including patients with septic shock at 31 sites. After excluding patients with end-stage kidney disease and missing data, we determined frequencies of the following clinical outcomes: AKI (defined within 24 h as Kidney Disease: Improving Global Outcomes stages 2 or 3 or stage 1 with tissue inhibitor of metalloproteinases-2 × insulin-like growth factor binding protein 7 value of > 2.0), CKD, and AKD (persistence of AKI at any stage on day 7 after enrollment) across four phenotypes. We performed multivariable logistic regression to assess the risk-adjusted association between development of AKI and AKD and phenotype.

**Results:**

Among 1,090 eligible patients, 543 patients (50%) had AKI. Across phenotypes, the frequencies of AKI varied, being highest in the δ and β phenotypes (78% and 71%, respectively) and the lowest in the α phenotype (26%; *P < .*001). AKD occurred most often in the δ phenotype (41%) and least often in the α phenotype (8%; *P < .*001). The highest frequencies of CKD and of AKI on CKD were found in the β phenotype (53% and 38% respectively; *P < .*001 for both). In the multivariable logistic regression models (α phenotype as reference), δ phenotype showed the strongest association with AKI (OR, 12.33; 95% CI, 7.81-19.47; *P < .*001) and AKD (OR, 9.18; 95% CI, 5.44-15.51; *P < .*001).

**Interpretation:**

The rates of AKI and AKD differed across clinical sepsis phenotypes and are more common among patients with phenotypes β and δ. Phenotype β showed a higher level of underlying CKD that predisposed patients to new AKI. The α and γ phenotypes showed lower frequencies of AKI and less progression to AKD.

## Graphical Abstract

**Figure:**
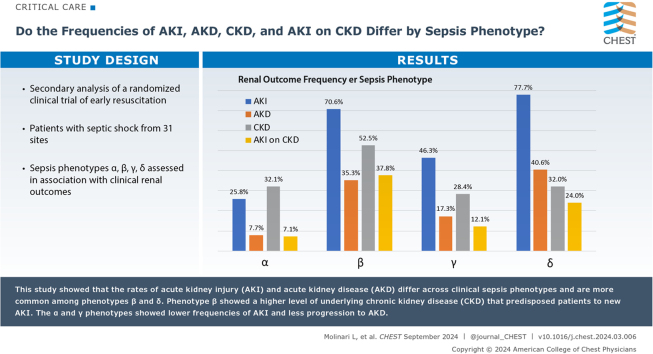

FOR EDITORIAL COMMENT, SEE PAGE 415

**Take-home Points**

**Study Question:** Do the frequencies of acute kidney injury, acute kidney disease, chronic kidney disease, and acute kidney injury on chronic kidney disease differ by the four clinical phenotypes for sepsis (α, β, γ, and δ)?

**Results:** The frequencies of acute kidney injury were the highest in the δ and β phenotypes and the lowest in the α phenotype. Acute kidney disease occurred most often in δ phenotype and least often in α phenotype. The highest frequencies of chronic kidney disease and of acute kidney injury on chronic kidney disease were found in the β phenotype.

**Interpretation:** Kidney diseases were more common in patients with phenotypes β and δ. Phenotype β showed a higher level of underlying chronic kidney disease that predisposed patients to new acute kidney injury, phenotype δ was more likely to progress to acute kidney disease, and phenotypes α and γ showed lower frequencies of acute kidney injury and less progression to acute kidney disease.

Acute kidney injury (AKI) is common among critically ill patients and increases the risk of morbidity and mortality.^1^, ^2^, ^3^ AKI is a broad term that includes many clinical syndromes, such as hepatorenal syndrome, cardiorenal syndrome, nephrotoxic syndrome, sepsis-associated syndrome, and urinary tract obstruction. These syndromes have different pathophysiologic mechanisms, biomarker signatures, and treatments.^4^^,^^5^ Sepsis is the most common cause of AKI in critically ill patients,^1^^,^^3^^,^^6^^,^^7^ and AKI is an important risk factor for the development of sepsis.^8^ AKI in the setting of sepsis is associated with higher risk of hospital mortality and length of hospitalization compared with other causes of AKI.^9^ Although the understanding of the pathophysiologic features of sepsis-associated AKI^10^ has increased, no specific therapies have been approved.^11^

Recently, four clinical phenotypes for sepsis were derived using machine learning on clinical and laboratory data in electronic health records.^12^ These phenotypes differ in patterns of organ dysfunction, prognosis, and response to treatment. The severity and occurrence of kidney dysfunction varied among phenotypes, but a deeper understanding of AKI, chronic kidney disease (CKD), or both across phenotypes is unknown. A better understanding of how these conditions relate to the clinical phenotypes could refine treatment strategies, tailoring them to a specific pattern of clinical features and organ damage.

To address this knowledge gap, we examined data from the Protocolized Care for Early Septic Shock (ProCESS) trial^13^ to evaluate: (1) the presence and distribution of AKI, acute kidney disease (AKD), CKD, and AKI on CKD across the four clinical phenotypes of sepsis; (2) the extent to which phenotypes are associated with AKI and AKD; and (3) the effect of kidney disease on survival according to phenotypes.

## Study Design and Methods

### Study Design and Patients

We performed a secondary analysis of the ProCESS trial.^13^^,^^14^ The ProCESS study was a multicenter randomized clinical trial (RCT) that enrolled 1,341 patients to compare alternative resuscitation strategies (protocol-based early goal-directed therapy, protocol-based standard therapy, and usual care) for patients with septic shock in 31 EDs in the United States. The study found no differences in 60-day mortality. An ancillary study to the ProCESS trial^14^ concluded that, although AKI was associated with adverse outcomes, AKI and its severity were not affected by the different resuscitation strategies (e-Appendix 1, “ProCESS trial and ProGReSS-AKI Study”). AKI was classified and staged at enrollment and daily based on maximum severity by either serum creatinine (sCr) or urine output (UO) criteria of Kidney Disease: Improving Global Outcomes (KDIGO)^15^ (e-Appendix 1, “AKI Staging and Reference Serum Creatinine”).

For each patient, we collected demographics, Charlson Comorbidity Index,^16^ severity scores at enrollment (Acute Physiology and Chronic Health Evaluation II^17^ and Sequential Organ Failure Assessment),^18^ sCr (at enrollment and daily), and UO (hourly for the first 72 h or until discharge from the ICU).^15^ Urine samples were obtained at enrollment and 6 h afterward and were assayed for tissue inhibitor of metalloproteinases-2 (TIMP-2) and insulin-like growth factor binding protein 7 (IGFBP7) with the NephroCheck test (Astute Medical, Inc.) according to the manufacturer’s specifications. We used the high-specificity cutoff of 2.0 (ng/mL)^2^/1,000 for the product of the two markers^19^ to stratify patients with KDIGO AKI stage 1 further and to select patients at higher risk of death or dialysis according to previous evidence.^20^^,^^21^ This retrospective study involving human participants to a clinical trial was conducted in accordance with the ethical standards of the institutional and national research committee and with the tenets of the 1964 Declaration of Helsinki. The Office of Human Research Protection at University of Pittsburgh approved this ancillary analysis conducted using deidentified data.

### Identification of Sepsis Phenotypes

Each patient from the ProCESS trial was assigned previously by Seymour et al^12^ to one of the four clinical phenotypes for sepsis (α, β, γ, or δ) (e-Appendix 1). The four phenotypes were derived and validated using unsupervised clustering methods that were applied to clinical variables associated with sepsis onset and outcome (eg, demographics, vital signs, markers of inflammation, and markers of organ dysfunction or injury); for each candidate variable, the most abnormal value recorded within the first 6 h of presentation was used.

### Outcomes

We evaluated AKI as the primary outcome, whereas as secondary outcomes were AKD, CKD, AKI on CKD, and mortality and survival up to 90 days from enrollment*.* AKI was defined as the presence of stage 2 or 3 disease using full KDIGO criteria (UO and sCr) within 24 h from enrollment. Given that KDIGO stage 1 disease together with urinary TIMP-2 × IGFBP7 values of > 2.0 (ng/mL)^2^/1,000 (stage 1B)^22^ have a similar outcome compared with stage 2 or 3 AKI,^20^ we included positive biomarker findings in stage 1 disease in our definition based on biomarker levels at 6 h from enrollment. If urinary TIMP-2 × IGFBP7 value at 6 h was missing, the value at enrollment was used in its place; if both TIMP-2 × IGFBP7 values were missing, then the patient was excluded from the primary analysis cohort. The choice of using first the measurements at 6 h and then using the one at enrollment only if the 6-h measurement was missing was based on previous findings that suggest a more informative role in sepsis-associated AKI for TIMP-2 × IGFBP7 values if tested at 6 to 12 h from sepsis diagnosis and treatment.^23^^,^^24^ AKD was defined only for patients with AKI as the presence of any AKI stage (including biomarker-negative stage 1) at 7 days from enrollment.^25^ If sCr or UO data were not available at day 7 to assess AKI and AKD, the most recent data closest to day 7 were used. Patients who died with AKI were included in AKD. Separately, we also evaluated AKD in the subgroup of patients who were still alive on day 7. CKD was defined as chart-reported history of kidney disease or reference estimated glomerular filtration rate of < 60 mL/min/1.73 m^2^. Reference estimated glomerular filtration rate was calculated using the Modification of Diet in Renal Disease study equation^26^ from reference sCr.^14^ AKI on CKD was the concomitant presence of the previously defined AKI and CKD. We also evaluated mortality and survival up to 90 days from enrollment as secondary outcomes and non-oliguric AKI, the use of nephrotoxic antibiotics, and the original infection site as exploratory risk factors (definitions provided in e-Appendix 1).

### Statistical Analysis

We assessed the distribution of the outcomes across the four phenotypes, we assessed the strength of the association between the phenotypes and AKI and AKD, and we assessed the effect of the phenotypes and the primary and secondary outcomes on 90-day survival. First, we used the Pearson χ^2^ test to compare the distribution of the outcomes and the exploratory risk factors across the four phenotypes. For pairwise comparison between phenotypes, we applied Bonferroni correction. Next, we performed multivariable logistic regression using the outcome AKI (in the entire cohort and separately in patients with and without CKD) or AKD as the dependent variable and using the phenotypes as the independent variable (as a nominal categorical variable). We adjusted the models for age, sex, weight, race, and comorbidities. Finally, we performed a log-rank test to assess difference in survival, censored at 90 days, among the four phenotypes and between the presence or absence of AKI*,* AKD, and CKD. We also ran four Cox proportional hazard models adjusting for age, sex, weight, race, and comorbidities with *P < .*10 at univariate analysis for mortality at 90 days. We used all-cause mortality, censored at 90 days, as the outcome and, as covariate or primary exposure, the phenotypes in the first model and AKI, AKD, and CKD in the second, third, and fourth models, respectively. We drew adjusted survival curves accordingly. We also performed an interaction analysis creating an interaction variable in the Cox proportional hazard model to test whether the effect of the AKI, AKD, and CKD on survival varied by sepsis phenotype, and so we built three other Cox models (models A, B, and C), entering as independent variables the phenotypes together with one of the outcomes at a time.

For complete case analysis, we performed a sensitivity analysis using multiple imputation by chained equation methodology to address missingness in variables such as TIMP-2 × IGFBP7 values at enrollment, age, lactate level, Sequential Organ Failure Assessment score, and weight. Predictive mean matching was performed by creating 25 iterations of different data sets. Analysis for missing variables before and after distribution was assessed. Then, we reran the statistical analysis used for the primary analysis. Details regarding missing data and the sensitivity analysis are provided in e-Appendix 1.

We used SPSS Statistics version 26 software (IBM Corp.) and the per-comparison significance was set at a two-tailed *P* values of < .05. We used STATA version 16.1 software (StataCorp LLC) to perform multiple imputations.

## Results

### Baseline Characteristics

Among 1,090 patients (e-Fig 1), 364 patients (33.4%) were in the α phenotype, 238 patients (21.8%) were in the β phenotype, 313 patients (28.7%) were in the γ phenotype, and 175 patients (16.1%) were in the δ phenotype at enrollment. Patents in the β phenotype were older (median age, 69 years; interquartile range [IQR], 57-79 years) than the α and γ phenotypes, and they had the highest Charlson Comorbidity Index (3; IQR, 2-6). Patients with δ phenotype had the highest Acute Physiology and Chronic Health Evaluation II score (median, 24; IQR 20-30) and Sequential Organ Failure Assessment score (10; IQR 8-12) (Table 1). The median TIMP-2 × IGFBP7 value was 0.38 (ng/mL)^2^/1,000 (interquartile range 0.13-1.47 (ng/mL)^2^/1,000) for the overall cohort, and 224 patients (20.6%) demonstrated a TIMP-2 × IGFBP7 value of > 2.0 (ng/mL)^2^/1,000 (Table 1).

**Table 1:** Baseline Characteristics of the Primary Analysis Cohort and Their Distributions Across the Four Clinical Phenotypes for Sepsis

Variable	Total (N = 1,090)	Phenotype			
		α (n = 364)	β (n = 238)	γ (n = 313)	δ (n = 175)
Age, y	61 (50-74)	57 (46-70)	69 (57-79)	60 (48-70)	63 (54-75)
Sex, male	610 (56)	194 (53.3)	141 (59.2)	166 (53)	109 (62.3)
Weight, kg	76 (64-91)	77 (64-92)	77 (66-92)	75 (63-91)	74 (64-90)
Race^a^					
White	758 (69.5)	258 (70.9)	174 (73.1)	217 (69.3)	109 (62.3)
Black	258 (23.7)	76 (20.9)	53 (22.3)	71 (22.7)	58 (33.1)
Other	74 (6.8)	30 (8.2)	11 (4.6)	25 (8)	8 (4.6)
Diabetes	359 (32.9)	94 (25.8)	104 (43.7)	109 (34.8)	52 (29.7)
Cardiovascular disease (total)^b^	703 (64.5)	208 (57.1)	191 (80.3)	203 (64.9)	101 (57.7)
Arterial hypertension	630 (57.8)	188 (51.6)	174 (73.1)	174 (55.6)	94 (53.7)
Congestive heart failure	120 (11)	32 (8.8)	47 (19.7)	23 (7.3)	18 (10.3)
Prior myocardial infarction	117 (10.7)	29 (8)	37 (15.5)	30 (9.6)	21 (12)
Cerebral vascular disease	114 (10.5)	24 (6.6)	32 (13.4)	26 (8.3)	32 (18.3)
Peripheral vascular disease	83 (7.6)	21 (5.8)	22 (9.2)	21 (6.7)	19 (10.9)
Chronic respiratory disease	250 (22.9)	78 (21.4)	68 (28.6)	69 (22)	35 (20)
Active cancer	197 (18.1)	50 (13.7)	68 (28.6)	56 (17.9)	23 (13.1)
Dementia	86 (7.9)	24 (6.6)	19 (8)	17 (5.4)	26 (14.9)
Liver cirrhosis	69 (6.3)	7 (1.9)	13 (5.5)	27 (8.6)	22 (12.6)
Ulcer disease	62 (5.7)	24 (6.6)	18 (7.6)	9 (2.9)	11 (6.3)
HIV infection	27 (2.5)	4 (1.1)	8 (3.4)	12 (3.8)	3 (1.7)
Charlson comorbidity index	2 (1-4)	1 (0-2)	3 (2-6)	2 (1-3)	2 (1-3)
APACHE II score	19 (15-25)	16 (12-20)	21 (18-26)	19 (16-23)	24 (20-30)
SOFA score	7 (4-9)	5 (3-7)	7 (5-10)	7 (4-9)	10 (8-12)
Lactate, mmol/L	4.4 (2.5-6)	4.3 (2.1-5.3)	3.6 (1.8-5.0)	4.3 (2.6-5.9)	6.7 (4.8-9.1)
Reference creatinine, mg/dL	1.0 (0.8-1.3)	1.0 (0.8-1.2)	1.1 (0.9-1.4)	1.0 (0.8-1.2)	1.0 (0.8-1.2)
TIMP-2 × IGFBP7 > 2.0 (ng/mL)^2^/1,000^c^	224 (20.6)	49 (13.5)	44 (18.5)	70 (22.4)	61 (34.9)
TIMP-2 × IGFBP7, (ng/mL)^2^/1000^c^	0.38 (0.13-1.47)	0.25 (0.08-0.78)	0.55 (0.16-1.42)	0.36 (0.13-1.69)	0.85 (0.23-3.06)

Data are presented as No. (%) or median (interquartile range). APACHE = Acute Physiology and Chronic Health Evaluation; IGFBP7 = insulin-like growth factor binding protein 7; SOFA = Sequential Organ Failure Assessment; TIMP-2 = tissue inhibitor metalloproteinases-2.

a White race corresponds to White/Caucasian; Black race corresponds to Black/African American; other race corresponds to Asian, American Indian or Native Alaskan, Native Hawaiian, or other Pacific Islander, unknown, or other.

b Presence of any among arterial hypertension, congestive heart failure, previous myocardial infarction, cerebral vascular disease, and peripheral vascular disease.

c TIMP-2 × IGFBP7 at 6 h from enrollment. For 91 patients, this value was missing and the value at enrollment was used in its place.

### Outcomes by Phenotype

AKI was present in 543 patients (50%); among them, 319 patients (29%) had stage 2 disease, 198 patients (18%) had stage 3 disease, and 26 patients (2%) had stage 1B disease (biomarker-positive stage 1).^22^ The β and δ phenotypes showed the highest frequencies of AKI (70.6% and 77.7%, respectively), followed by the γ phenotype (46.3%) and the α with the lowest frequency of AKI (25.8%; *P < .*001 overall). Late AKI (manifesting 24-48 h after enrollment, but not present within 0-24 h) was rare (31 patients [2.8%]) and was seen mainly in α and γ phenotypes (e-Table 1). AKD was present in 237 of 1,090 patients (21.7%). The highest frequencies were observed for δ and β phenotypes (40.6% and 35.3%, respectively) (Table 2). Considering only survivors at 7 days, among the 543 patients with AKI, 458 patients (84.3%) survived ≥ 7 days. Of these 458 survivors, 169 patients (36.9%) had AKD. The highest frequencies of AKD among survivors at 7 days again were for the δ and β phenotypes (45.8% and 44.0%, respectively), followed by the γ phenotype (29.5%) and α phenotype (25.0%; *P = .*002 overall). CKD was present in 35.5% of all the patients, but this percentage was highest (52.5%) in the β phenotype group (*P < .*05 for all pairwise comparisons). AKI on CKD were present together in 18% of all the patients, and this percentage again was higher for patients in the β phenotype group (37.8%). All-cause mortality at 90 days was 28.9%. Mortality at 90 days varied across all the phenotypes (*P < .*001 overall), with the lowest frequency in the α phenotype (12.4%) and the highest in the δ phenotype (45.7%) (Table 2). Acute renal replacement therapy was uncommon in this cohort (42 patients [3.4%]); however, more than one-third of these (17 patients) were in the δ phenotype group (e-Table 1).

**Table 2:** Distribution of the Outcomes and the Exploratory Risk Factors Across the Four Clinical Phenotypes for Sepsis

Variable	Total (N = 1,090)	Phenotype				*P* Value
		α (n =364)	β (n =238)	γ (n =313)	δ (n =175)	
Primary outcome						
AKI	543 (49.8)	94 (25.8)^a^	168 (70.6)^b^	145 (46.3)^c^	136 (77.7)^b^	< .001
Secondary outcomes						
AKD	237 (21.7)	28 (7.7)^a^	84 (35.3)^b^	54 (17.3)^c^	71 (40.6)^b^	< .001
CKD	387 (35.5)	117 (32.1)^a^	125 (52.5)^b^	89 (28.4)^a^	56 (32)^a^	< .001
AKI on CKD	196 (18)	26 (7.1)^a^	90 (37.8)^b^	38 (12.1)^a^	42 (24)^c^	< .001
AKI not present/CKD not present	356 (32.7)	179 (49.2)^a^	35 (14.7)^b^	117 (37.4)^c^	25 (14.3)^b^	...
AKI not present/ CKD present	191 (17.5)	91 (25)^a^	35 (14.7)^b^	51 (16.3)^b^	14 (8)^b^	...
AKI present/ CKD not present	347 (31.8)	68 (18.7)^a^	78 (32.8)^b^	107 (34.2)^b^	94 (53.7)^c^	...
Mortality at 90 d	315 (28.9)	45 (12.4)^a^	89 (37.4)^b^^,^^c^	101 (32.3)^b^	80 (45.7)^c^	< .001
Exploratory risk factors						
Nonoliguric AKI	265 (24.3)	38 (10.4)^a^	93 (39.1)^b^	73 (23.3)^c^	61 (34.9)^b^	< .001
Nephrotoxic antibiotic use	921 (84.5)	299 (82.1)^a^	200 (84)^a^	269 (85.9)^a^	153 (87.4)^a^	.36
Infection site^d^						.03
Pneumonia	357 (32.8)	125 (34.3)^a^	57 (23.9)^b^	117 (37.4)^a^	58 (33.1)^a^^,^^b^	
Urosepsis	243 (22.3)	74 (20.3)	58 (24.4)	72 (23)	39 (22.3)	
Unknown	136 (12.5)	44 (12.1)	27 (11.3)	42 (13.4)	23 (13.1)	
Intraabdominal	135 (12.4)	41 (11.3)	36 (15.1)	35 (11.2)	23 (13.1)	
Skin and soft tissues	79 (7.2)	26 (7.1)	23 (9.7)	20 (6.4)	10 (5.7)	
Others	76 (7)	26 (7.1)	16 (6.7)	18 (5.8)	16 (9.1)	
None	25 (2.3)	14 (3.8)	8 (3.4)	1 (0)	3 (1.7)	
Catheter related	22 (2)	5 (1.4)	8 (3.4)	7 (2.2)	2 (1.1)	
CNS	10 (0.9)	7 (1.9)	2 (0.8)	1 (0)	1 (0.6)	
Endocarditis	7 (0.6)	2 (0.5)	3 (1.3)	2 (0.6)	1 (0)	

Data are presented as No. (%). If overall *P < .*05, pairwise comparisons between phenotypes were performed using Bonferroni correction. AKD = acute kidney disease; AKI = acute kidney injury; CKD = chronic kidney disease.

a,b,c Values in the same row not sharing the same superscript letter are significantly different at adjusted *P <* .05.

d See e-Appendix 1 for definitions.

Nonoliguric AKI was seen in 265 patients (24.3%; 48.8% of those with AKI) and the distribution of nonoliguric AKI was different across the four phenotypes (*P < .*001 overall). The highest frequencies of nonoliguric AKI were found in the β phenotype group (39.1%; 55.4% of total patients with AKI in the β phenotype group). No difference across the phenotypes was found regarding the use of nephrotoxic antibiotics (at least one for at least 1 day; *P = .*36); although the overall distribution of the infection site was different across the phenotypes (*P = .*03 overall), no differences were found in the frequencies of urosepsis and only a slightly lower prevalence of pneumonia was found in the β phenotype (23.9%) compared with the α and γ phenotypes (34.3% and 37.4%, respectively).

### Associations Between Phenotypes and Primary Outcomes

Using the α phenotype as reference, the association of δ phenotype for AKI in the entire cohort showed an adjusted OR of 12.33 (95% CI, 7.81-19.47; *P < .*001), in patients without CKD showed an OR of 11.93 (95% CI, 6.82-20.87; *P < .*001), and in patient with CKD showed an OR of 12.91 (95% CI, 5.67-29.36; *P < .*001). The association of δ phenotype for AKD showed an OR of 9.18 (95% CI, 5.44-15.51; *P < .*001). Patients with the β phenotype showed an OR of 8.32 (95% CI, 5.55-12.48; *P < .*001) for AKI in the entire cohort, an OR of 12.38 (95% CI, 6.19-24.79; *P < .*001) in patients without CKD, and an OR of 12.38 (95% CI, 6.19-24.79; *P < .*001) in patients with CKD. However, when comparing β phenotype (as reference) with δ phenotype, ORs were not significant except for AKI in patients without CKD (OR, 1.92; 95% CI, 1.03-3.60; *P = .*04). The γ phenotype was associated more strongly than the α phenotype with all outcomes, but always less than the β and δ phenotypes (Table 3).

**Table 3:** Logistic Regression Models Showing the Association of the Clinical Phenotypes for Sepsis With AKI (Evaluated in the Entire Primary Analysis Cohort, in Patients Without CKD, and in Patient With CKD) and AKD

Model	Primary Analysis	
Dependent variable	Adjusted^a^ OR (95% CI)	*P* Value
AKI entire cohort (N = 1,090)		
Phenotype β (reference, α)	8.32 (5.55-12.48)	**< .001**
Phenotype γ (reference, α)	2.84 (2.01-3.99)	**< .001**
Phenotype δ (reference, α)	12.33 (7.81-19.47)	**< .001**
Phenotype β (reference, γ)	2.94 (2.00-4.31)	**< .001**
Phenotype δ (reference, γ)	4.35 (2.80-6.76)	**< .001**
Phenotype δ (reference, β)	1.48 (0.92-2.39)	.11
AKI in patients without CKD (n = 703)		
Phenotype β (reference, α)	6.20 (3.66-10.51)	**< .001**
Phenotype γ (reference, α)	2.70 (1.79-4.05)	**< .001**
Phenotype δ (reference, α)	11.93 (6.82-20.87)	**< .001**
Phenotype β (reference, γ)	2.30 (1.39-3.81)	**.001**
Phenotype δ (reference, γ)	4.42 (2.57-7.61)	**< .001**
Phenotype δ (reference, β)	1.92 (1.03-3.60)	**.04**
AKI in patients with CKD (n = 387)		
Phenotype β (reference, α)	12.38 (6.19-24.79)	**< .001**
Phenotype γ (reference, α)	3.16 (1.65-6.04)	**.001**
Phenotype δ (reference, α)	12.91 (5.67-29.36)	**< .001**
Phenotype β (reference, γ)	3.92 (2.04-7.56)	**< .001**
Phenotype δ (reference, γ)	4.09 (1.84-9.09)	**.001**
Phenotype δ (reference, β)	1.04 (0.47-2.30)	.92
AKD (N = 1,090)		
Phenotype β (reference, α)	7.63 (4.55-12.80)	**< .001**
Phenotype γ (reference, α)	2.98 (1.79-4.97)	**< .001**
Phenotype δ (reference, α)	9.18 (5.44-15.51)	**< .001**
Phenotype β (reference, γ)	2.56 (1.68-3.91)	**< .001**
Phenotype δ (reference, γ)	3.08 (1.99-4.77)	**< .001**
Phenotype δ (reference, β)	1.20 (0.78-1.85)	.40

Statistical significance indicated by boldface. AKD = acute kidney disease; AKI = acute kidney injury; CKD = chronic kidney disease.

a ORs were adjusted for age, sex, weight (after logarithm transformation), race, diabetes, arterial hypertension, congestive heart failure, chronic respiratory disease, and cancer.

### Survival and Interaction Analysis

**Figure 1:**
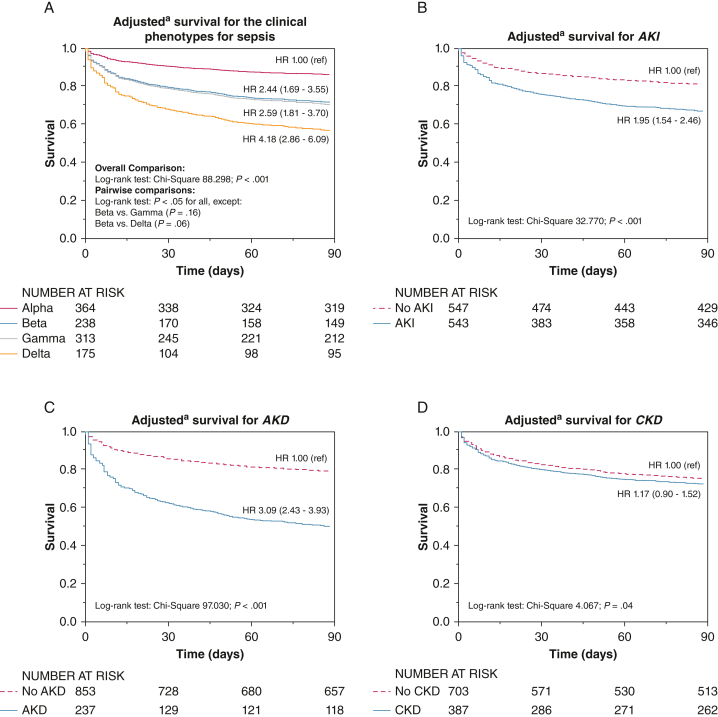
Covariate-adjusted survival curves at 90 d obtained from Cox proportional hazard models. A, Survival curves for each of the four sepsis phenotypes. B, Suvival curves for AKI vs no AKI. C, Survival curves for AKD vs no AKD . D, Survival curves for CKD vs no CKD. ^a^Hazard ratios (95% CIs) and survival curves in each panel were obtained from different Cox proportional hazard models. In each model, the variable shown in the figure was evaluated separately only with the other adjusting covariates. The adjusting covariates were: age, sex, weight (after logarithm transformation), race, diabetes, and comorbidities with significance of *P < .*10 at univariate analysis (not shown) for mortality at 90 days. The comorbidities entered in all the above models were: cardiovascular disease (presence of any between arterial hypertension, history of congestive heart failure, prior myocardial infarction, cerebral vascular disease, or peripheral vascular disease), cancer, dementia, liver cirrhosis, and HIV infection. AKD = acute kidney disease; AKI = acute kidney injury; CKD = chronic kidney disease; HR = hazard ratio; ref = reference.

Using α phenotype as reference, the adjusted hazard ratios (HRs) for death within 90 days were 2.44 (95% CI, 1.69-3.55; *P < .*001) for β phenotype, 2.59 (95% CI, 1.81-3.70; *P < .*001) for γ phenotype, and 4.18 (95% CI, 2.86-6.09; *P* < .001) for δ phenotype (Fig 1A). The HRs for δ phenotype were 1.71 (95% CI, 1.24-2.35; *P = .*001) and 1.62 (95% CI, 1.19-2.19; *P = .*002) using β and γ phenotypes as a reference, respectively, whereas the HR for γ phenotype (with reference β phenotype) was 1.06 (95% CI, 0.79-1.42; *P = .*72). The presence of AKI was associated with lower survival (*P < .*001, log-rank test) with an adjusted HR of 1.95 (95% CI, 1.54-2.46; *P < .*001) (Fig 1B), and so was the presence of AKD (*P < .*001, log-rank test) with an adjusted HR of 3.09 (95% CI, 2.43-3.93; *P < .*001) (Fig 1C). For CKD, the log-rank test was significant (*P < .*04), but the adjusted HR was not (1.17; 95% CI, 0.90-1.52; *P = .*24) (Fig 1D, e-Appendix 1, “Proportional Hazards Assumption Testing for Cox Models”). Table 4 showed different Cox proportional hazard models for different combinations of the phenotypes and renal outcomes. No significant interaction (*P* > 0.05 for all) was present among phenotypes, renal outcomes, and survival at 90 days.

**Table 4:** Cox Proportional Hazard Models for Mortality Within 90 Days With Different Combinations of the Phenotypes and Renal Outcomes

Variable	Model A		Model B		Model C	
	HR (95% CI)^a^	*P* Value	HR (95% CI)^a^	*P* Value	HR (95% CI)^a^	*P* Value
Phenotype β (reference, α)	2.06 (1.40-3.04)	**< .001**	1.83 (1.25-2.69)	**.002**	2.38 (1.63-3.48)	**< .001**
Phenotype γ (reference, α)	2.35 (1.63-3.37)	**< .001**	2.36 (1.65-3.38)	**< .001**	2.60 (1.82-3.71)	**< .001**
Phenotype δ (reference, α)	3.42 (2.30-5.08)	**< .001**	3.19 (2.17-4.70)	**< .001**	4.15 (2.85-6.06)	**< .001**
Phenotype γ (reference, β)	1.14 (0.85-1.53)	.39	1.29 (0.96-1.74)	.10	1.09 (0.81-1.48)	.58
Phenotype δ (reference, β)	1.66 (1.21-2.28)	**.002**	1.74 (1.27-2.40)	**.001**	1.74 (1.26-2.41)	**.001**
Phenotype δ (reference, γ)	1.46 (1.07-1.98)	**.02**	1.35 (0.99-1.84)	.06	1.60 (1.18-2.17)	**.003**
AKI^b^	1.51 (1.17-1.94)	**.001**	...	...	...	...
AKD^b^	...	...	2.68 (2.08-3.44)	**< .001**	...	...
CKD^b^	...	...	...	...	1.13 (0.87-1.47)	.37

Statistical significance indicated by boldface. AKD = acute kidney disease; AKI = acute kidney injury; CKD = chronic kidney disease; HR = hazard ratio.

a Adjusted for age, sex, weight (after logarithm transformation), race, diabetes, and comorbidities with significance at *P <* .10 in univariable analysis for mortality at 90 d (not shown). The comorbidities entered in all models were: cardiovascular disease (presence of any between arterial hypertension, history of congestive heart failure, prior myocardial infarction, cerebral vascular disease, or peripheral vascular disease), cancer, dementia, liver cirrhosis, and HIV infection.

b No interaction with the phenotypes (*P* > .05).

### Sensitivity Analysis

After accounting for missing data, we included 1,243 patients (e-Fig 1), of whom 417 patients (33.5%) were in the α phenotype group, 277 patients (22.3%) were in the β phenotype group, 343 patients (27.6%) were in the γ phenotype group, and 206 patients (16.6%) were in the δ phenotype group. The sensitivity analysis (e-Tables 2-7, e-Fig 2) showed that overall results were consistent with the primary analysis reported herein.

## Discussion

The highest frequencies of AKI were found in the δ and β phenotypes, and in both phenotypes, the presence of AKI was associated with a higher risk of AKD. The results from Seymour et al^12^ regarding different biomarker domains showed that the δ phenotype is associated with hyperinflammation and abnormal coagulation. These may be the leading mechanisms causing sepsis-associated multiple organ injury and in particular AKI in this phenotype.^6^ However, the patients in the β phenotype group showed the highest frequency of comorbidities, and more than one-half of the patients with AKI at presentation had underlying CKD. The reduced renal reserve caused by CKD and older age presumably is one of the major reasons for increased susceptibility to AKI,^27^ and both conditions are typical of the β phenotype. Notably, a larger proportion of patients with AKI were nonoliguric in the β phenotype. Although nonoliguric AKI often is a nonspecific indicator of drug-associated AKI, exposure to nephrotoxins did not vary by phenotype.

This work lays a potential foundation to inform both clinical trials and clinical decision-making for sepsis-associated kidney disease. In the future, we may be able to determine a patient’s phenotype in the first hours from sepsis diagnosis using common and rapidly available clinical variables, which will allow for better alignment of treatment strategies as well as providing useful early prognostic information by phenotype. For instance, therapies to prevent AKI or to reduce severity may be more effective in patients of the δ phenotype than in patients of the α phenotype, where the frequencies of AKI and AKD are quite low.

The use of clinical or biomarker phenotyping to guide the implementation of kidney-sparing bundles of care recommended by KDIGO guidelines^15^ maximizes the potential impact on outcomes. For instance, the protective impact of implementing these guidelines in patients with phenotypes that portray a higher risk for AKI and AKD (eg, β and δ phenotypes) is likely to be larger than when implemented in patients with lower risk. The application of kidney-sparing bundles guided by AKI risk profiles using biomarkers has been tested in RCTs of patients undergoing surgery with promising results.^28^^,^^29^ A similar strategy may be necessary in sepsis to maximize the possibility of affecting outcomes given the wide heterogeneity of the syndrome. Following this logic, the Limiting AKI Progression in Sepsis trial^30^^,^^31^ was designed to evaluate the application of these bundles guided by serial measurements of TIMP-2 × IGFBP7 in patients with sepsis who did not have KDIGO stage 2 or 3 disease AKI at enrollment.

Clinical phenotypes for sepsis also may be used to enrich future RCTs for patients with underlying pathologic mechanisms addressed by specific treatments. For instance, the γ and δ phenotypes have shown the highest levels of inflammatory markers and cytokines.^12^ Thus, selecting patients within these phenotypes would provide an enrichment strategy for studies testing medications that reduce inflammation such as human recombinant alkaline phosphatase, maximizing the potential impact of the therapy on reducing organ dysfunction (in particular kidney dysfunction) and mortality.^32^ Early studies showed alkaline phosphatase as a promising treatment to prevent sepsis-associated AKI.^33^^,^^34^ Although recent trials failed to meet the primary end points,^35^^,^^36^ it is possible that the inclusion of all phenotypes may have washed out the positive effect that only certain phenotypes would have demonstrated. Similarly, hemoadsorption therapy could be more effective in patients of the δ phenotype given the higher levels of inflammatory cytokines.^37^

Importantly, we did not find evidence that AKI (or AKD) impacts survival from sepsis differently across phenotypes. AKI remains a powerful predictor of mortality in sepsis independent of the underlying phenotype. As such, interventions to reduce AKI occurrence and severity in sepsis remain important regardless of the phenotype.

Our study has important limitations. First, it is a retrospective analysis using data from an RCT; although this allowed us to evaluate a large cohort with detailed outcome data, some data were missing (biomarkers, UO, and sCr after the first few days). Although the results of the sensitivity analysis using imputation were consistent with the primary analysis, it is still possible that some residual bias remained. Second, we were able to evaluate only survival as our outcome because data regarding other outcomes such as the need for dialysis and persistent kidney dysfunction were collected only over the first week. Third, the process of derivation and validation of the phenotypes, among different variables, including sCr, age, sex, and comorbidities, again was used in our analysis and models. This could have led to potential bias, in particular regarding sCr because it is also the main determinant for AKI definition and staging according to KDIGO criteria. However, in this study, we sought to distinguish between the different forms of kidney disease (AKI, AKD, and CKD) that were not considered in the derivation of the phenotype. Fourth, we defined AKD according to the criteria of Chawla et al^25^ as AKI not resolving at day 7 or the last day available. Because our statistical analysis plan was finalized, a new definition of AKD has been proposed that includes patients who do not first demonstrate AKI,^38^ and our analysis did not include these patients. Moreover, we did not consider other recovery phenotypes from AKI (eg, early sustained reversal, relapse, and so on).^39^ Fifth, because the reference creatinine was determined in the parent study and before race-free equations for estimated glomerular filtration rate, the Modification of Diet in Renal Disease study equation was used. However, little difference exists for patients without underlying CKD, and only in these patients was baseline creatinine estimated.

## Interpretation

Although AKI is common in patients with sepsis, important differences exist across the recently described phenotypes α, β, γ, and δ. AKI was shown to be most common in patients with δ and β phenotype, with patients of the δ phenotype exhibiting the highest frequencies of AKD, whereas patients of the β phenotype showed the highest frequencies of CKD and AKI on CKD.

## Funding/Support

The data for this project were obtained thanks to the following funding sources: the ProCESS trial (ClinicalTrials.gov Identifier: NCT00510835) funded by 10.13039/100000057National Institute of General Medical Sciences, National Institutes of Health [Grant P50GM076659]; the Goal-directed Resuscitation of Septic Shock to Prevent Acute Kidney Injury study, funded by the 10.13039/100000062National Institute of Diabetes and Digestive and Kidney Diseases, National Institutes of Health [Grant R01DK083961]; and the Sepsis Endotyping in Emergency Care project, funded by the 10.13039/100000002National Institutes of Health [Grant R35GM119519, with additional support from R21GM144851 and K08GM117310].

## Financial/Nonfinancial Disclosures

The authors have reported to *CHEST* the following: H. G. discloses consulting fees from Trilinear Bioventures, 10.13039/100004336Novartis, and AclRx; research grants from 10.13039/100004702Baxter and bioMérieux; and speaker fees from the National Kidney Foundation of Arizona and bioMérieux. C. W. S. discloses consulting fees from Octapharma, Inotrem, and Beckman Coulter. J. A. K. discloses research support and consulting fees from 10.13039/100009777Astute Medical and bioMérieux and is currently a full-time employee of Spectral Medical. None declared (L. M., G. D. R.-P., P. P., A. S., J. C. M., J. K.).

## Supplementary Data

e-Online Data
