# Supplementary material for: Distribution of Acute and Chronic Kidney Disease Across Clinical Phenotypes for Sepsis
Source: Chest. 2024 Mar 8;166(3):480–90. doi: 10.1016/j.chest.2024.03.006 (PMC11443243; doi:10.1016/j.chest.2024.03.006)
Supplement: e-Online Data [file mmc1.docx]

**SUPPLEMENTAL MATERIAL**

**Distribution of Acute and Chronic Kidney Disease across Clinical Phenotypes for Sepsis**

**Authors:**

Luca Molinari, MD;^1,2,3^ Gaspar Del Rio-Pertuz, MD;^2,4^ Priyanka Priyanka, BAMS MPH;^2^

Ali Smith, BA;^1^ Joseph C. Maggiore, BSE;^5^ Jason Kennedy, MS;^1^

Hernando Gomez, MD MPH;^1,2^ Christopher W. Seymour, MD MSc;^1^ John A. Kellum, MD;^1,2^

for the ProCESS and ProGReSS-AKI Investigators*

**Affiliations:**

^1^ Department of Critical Care Medicine, University of Pittsburgh, Pittsburgh, PA.

^2^ Center for Critical Care Nephrology, University of Pittsburgh, Pittsburgh, PA.

^3^ Department of Translational Medicine, Università degli Studi del Piemonte Orientale, Novara, Italy.

^4^ Division of Cardiology, Department of Medicine, University of Minnesota, Minneapolis, MN.

^5^ Department of Developmental Biology, University of Pittsburgh, Pittsburgh, PA.

*A complete list of ProCESS and ProGReSS-AKI Investigators is available at <https://crisma.upmc.com/progressakistudy> and in the Supplemental Material (page 3).

**INDEX OF CONTENTS:**

[ProCESS and ProGReSS-AKI Investigators 3](#_Toc160541762)

[ProCESS TRIAL and ProGReSS-AKI STUDY 4](#_Toc160541763)

[AKI STAGING AND REFERENCE SERUM CREATININE 5](#_Toc160541764)

[THE CLINICAL PHENOTYPES FOR SEPSIS 6](#_Toc160541765)

[DEFINITIONS OF EXPLORATORY RISK FACTORS 7](#_Toc160541766)

[MISSING DATA 8](#_Toc160541767)

[SENSITIVITY ANALYSIS 10](#_Toc160541768)

[**Methods** 10](#_Toc160541769)

[**Results** 11](#_Toc160541770)

[SUPPLEMENTAL TABLES 15](#_Toc160541771)

[**e-Table 1**. Late AKI and renal replacement therapy by phenotype. 15](#_Toc160541772)

[**e-Table 2.** Pre-imputation summary 16](#_Toc160541773)

[**e-Table 3.** Post-imputation summary 16](#_Toc160541774)

[**e-Table 4.** Baseline characteristics of the sensitivity analysis cohort and their distribution across the four clinical phenotypes for sepsis. 17](#_Toc160541775)

[**e-Table 5.** Distribution of the outcomes across the four clinical phenotypes for sepsis in the sensitivity analysis cohort. 18](#_Toc160541776)

[**e-Table 6.** Logistic regression models showing the association of the clinical phenotypes for sepsis with AKI (evaluated in the whole cohort, in patients without CKD and in patient with CKD) and AKD in the primary and sensitivity analysis cohort. 19](#_Toc160541777)

[**e-Table 7**. Cox proportional hazard models for death within 90 days with different combinations of the phenotypes and renal outcomes in the sensitivity analysis cohort. 20](#_Toc160541778)

[SUPPLEMENTAL FIGURES 21](#_Toc160541779)

[**e-Figure 1:** Study Flow. 21](#_Toc160541780)

[**e-Figure 2:** Covariate-adjusted survival curves at 90 days obtained from Cox proportional hazard models in the sensitivity analysis. 22](#_Toc160541781)

[PROPORTIONAL HAZARDS ASSUMPTION TESTING FOR COX MODELS 23](#_Toc160541782)

SUPPLEMENTAL MATERIAL REFERENCES 25

**ProCESS and ProGReSS-AKI Investigators**

**ProGReSS-AKI Investigators and Staff:** Derek C. Angus, Lakhmir S. Chawla, David T. Huang, Christopher Keener, John A. Kellum, Nicole Lucko, Paul M. Palevsky, Francis Pike, Kai Singbartl, Ali Smith, Donald M. Yealy, Sachin Yende.

**ProCESS Coordinating Center**: Derek C. Angus, Amber E. Barnato, Tammy L. Eaton, Elizabeth Gimbel, David T. Huang, Christopher Keener, John A. Kellum, Kyle Landis, Francis Pike, Diana K. Stapleton, Lisa A. Weissfeld, Michael Willochell, Kourtney A. Wofford, Donald M. Yealy.

**Recruiting Centers**: (Site Principal Investigators are listed in Italics) - Advocate Christ Medical Center, Oak Lawn, IL - *Erik Kulstad, Hannah Watts*. Allegheny General Hospital, Pittsburgh, PA - *Arvind Venkat*. Brigham and Women’s Hospital, Boston, MA - *Peter C. Hou, Anthony Massaro*, Siddharth Parmar. Duke University Medical Center, Durham, NC - *Alexander T. Limkakeng, Jr*. East Carolina University, Greenville, NC - Kori Brewer, *Theodore R. Delbridge*, Allison Mainhart. George Washington University Medical Center, Washington, DC - *Lakhmir S. Chawla*. Hennepin County Medical Center, Minneapolis, MN - *James R. Miner*. Intermountain Medical Center, Murray, UT - *Todd L. Allen, Colin K. Grissom*, Los Angeles County + USC Medical Center, Los Angeles, CA - *Stuart Swadron*. Louisiana State University Health Sciences Center, Shreveport, LA - *Steven A. Conrad*. Maricopa Medical Center, Phoenix, AZ - *Richard Carlson, Frank LoVecchio*. Massachusetts General Hospital, Boston, MA - *Ednan K. Bajwa, Michael R. Filbin*. Blair A. Parry. Methodist Research Institute, Indianapolis, IN - *Timothy J. Ellender*. North Shore University Hospital, Manhasset, NY - *Andrew E. Sama*. Norwalk Hospital, Norwalk, CT - *Jonathan Fine*. Penn State Hershey College of Medicine, Hershey, PA - Soheil Nafeei, *Thomas Terndrup, Margaret Wojnar*. Stanford University School of Medicine, Stanford, CA - *Ronald G. Pearl*. Summa Health System, Akron, OH - *Scott T. Wilber*. SUNY Downstate Medical Center, Brooklyn, NY - *Richard Sinert*. Tampa General Hospital, Tampa, FL - *David J. Orban*, Jason W. Wilson. Temple University Hospital, Philadelphia, PA - *Jacob W. Ufberg*. UC Davis Medical Center, Sacramento, CA - *Timothy Albertson, Edward A. Panacek*. University Medical Center Brackenridge, Austin, TX - *Sohan Parekh*. UPMC Presbyterian/Shadyside, Pittsburgh, PA - *Scott R. Gunn*, Jon S. Rittenberger, *Richard J. Wadas*. University of Alabama at Birmingham, Birmingham, AL - Andrew R. Edwards, Matthew Kelly, *Henry E. Wang*, University of Arkansas for Medical Sciences, Little Rock, AR - *Talmage M. Holmes*. University of Maryland at Baltimore, Baltimore, MD - *Michael T. McCurdy*. University of Minnesota Medical Center, Fairview, MN - *Craig Weinert*. University of Utah Health Sciences Center, Salt Lake City, UT - *Estelle S. Harris*. Vanderbilt University Medical Center, Nashville, TN - *Wesley H. Self*, Diane Dubinski. Washington Hospital Center, Washington, DC - *Carolyn A. Phillips*, Ronald M. Migues.

See also https://crisma.upmc.com/progressakistudy/processteam.asp?logged=0 or supplemental material (Supplement 1) from “*ProCESS Investigators, Yealy DM, Kellum JA, Huang DT, Barnato AE, Weissfeld LA, Pike F, Terndrup T, Wang HE, Hou PC, LoVecchio F, Filbin MR, Shapiro NI, Angus DC. A randomized trial of protocol-based care for early septic shock. N Engl J Med. 2014 May 1;370(18):1683-93. doi: 10.1056/NEJMoa1401602. Epub 2014 Mar 18. PMID: 24635773; PMCID: PMC4101700.”*

**ProCESS TRIAL and ProGReSS-AKI STUDY**

ProCESS^1^ was a multicenter, randomized clinical trial of three different resuscitation strategies in patients with septic shock that enrolled 1,341 patients to compare alternative resuscitation strategies (protocol-based early goal-directed therapy, protocol-base standard therapy, and usual care) for patients with septic shock in 31 emergency departments in the United States. All the sites were academic hospitals and to be eligible they had to have more than 40,000 emergency department visits per year and use serum lactate levels as the method for screening for cryptogenic shock. The sites had to follow the ongoing Surviving Sepsis Campaign guidelines^2^ for the non-resuscitation aspects but must not have routine resuscitation protocols for septic shock and must not routinely use continuous Scvo_2_ catheters. Patients were recruited in the emergency department if sepsis was suspected according to the treating physician, they were at least 18 years of age, they met two or more criteria for systemic inflammatory response syndrome^3^ and they had refractory hypotension or a serum lactate level of 4 mmol/L or higher. The trail did not find any difference in 60-day, 90-day and 1-year mortality or in the need of organ support between the different resuscitation strategies proposed.

Kellum and colleagues in a follow-up ancillary study (ProGReSS-AKI)^4^ specifically evaluated if the presence and the severity of acute kidney injury (AKI) were affected by the different resuscitation strategies. No association was found between different resuscitation strategies and development of acute kidney injury (AKI) and its severity in a subgroup of 1243 patients resulted after excluding patients for whom the assessment of AKI was impossible or unreliable, such as patients with end-stage kidney disease (chronic dialysis not related to the current sepsis episode), reference serum creatinine ≥4 mg/dL, or missing enrollment serum creatinine (see e-Figure 1).

**AKI STAGING AND REFERENCE SERUM CREATININE**

In ProGReSS-AKI study^4^ and in our study, AKI was classified and staged at enrollment and daily based on maximum severity by either serum creatinine (sCr) or urine output (UO) criteria by Kidney Disease: Improving Global Outcomes (KDIGO).^5^ We only assigned an AKI stage by urine output criteria if the urine output values were recorded. Missing urine output was not imputed and did not contribute to staging for patients.

Reference sCr was used for staging AKI and it was based on baseline (preadmission to hospital, historical) if known. In patients with no known baseline sCr by history we used KDIGO methodology to estimate the baseline based on the patient’s demographics using the Modification of Diet in Renal Disease (MDRD) equation.^6^ We only estimated baseline creatinine in this way in patients without a history of chronic kidney disease, again as per the guideline. In all cases the baseline was used as reference sCr, if known, otherwise the lowest value between estimated baseline and admission (to hospital) was used as the reference sCr.

Reference sCr was available for all patients. Baseline creatinine was missing for 679 (54.6%) patients in the whole cohort of 1,243 patients or for 588 (53.9%) patients in our primary analysis cohort of 1090 patients. Admission creatinine was missing for 55 (4.4%) out of 1243 patients or 41 (3.8%) out of 1090 patients. A total of 35 patients out of 1243 (or 25/1090) were missing both baseline and admission creatinine, and in these cases, we used the enrollment creatinine value obtained shortly after admission.

**THE CLINICAL PHENOTYPES FOR SEPSIS**

The four phenotypes have been derived and validated using unsupervised clustering methods that were applied to clinical variables associated with sepsis onset and outcome (e.g. demographics, vital signs, markers of inflammation, markers of organ dysfunction or injury); for each candidate variable the most abnormal value recorded within the first 6 hours of presentation was used. This work was part of a project by Seymour and colleagues.^7^

For the derivation and validation of the phenotypes the authors used 3 observational cohorts and 3 randomized clinical trial (RCT) cohorts.

The 3 observational cohorts were:

1. the SENECA derivation cohort was drawn from electronic health record data of adults who met sepsis criteria within the first 6 hours of presentation to emergency department in the UPMC health care system, during 2010 to 2012;
2. the SENECA validation cohort was drawn from electronic health record data of adults who met sepsis criteria within the first 6 hours of presentation to emergency department in the UPMC health care system, during 2013 to 2013;
3. the GenIMS study that was a multicenter prospective cohort of patients with severe community acquired pneumonia recruited from 4 regions in the United States.

The 3 RCTs were all studies involving patients with sepsis or septic shock:

1. ACCES compared eritoran (a highly specific myeloid differentiation protein 2 antagonist that inhibits toll-like receptor 4) vs placebo in a total of 1961 patients with severe sepsis enrolled in 197 intensive care units in 6 continents;^8^
2. PROWESS compared activated protein C vs placebo in a total of 1690 patients with severe sepsis at 164 sites in 11 countries ^9^
3. ProCESS compared early goal-directed vs alternative resuscitation approaches in a total of 1341 patients with septic shock at 31 sites in the United States.^1^

After the process of derivation and validation of the phenotypes^7^, each patient in each cohort was assigned to one of the 4 phenotypes. For our study we used the assignment for the ProCESS cohort.

**DEFINITIONS OF EXPLORATORY RISK FACTORS**

*Non-oliguric AKI*

*Non-oliguric AKI* was defined as the presence of *AKI* (as previously defined) but associated with KDIGO UO criteria for AKI not fulfilled or corresponding to stage 1 at maximum (UO <0.5 mL/kg/h for only 6-12 hours within the first 24 hours from enrollment).

*Nephrotoxic antibiotics and antiviral drugs*

We evaluated the use of acyclovir, amikacin, cefotaxime, ceftazidime, cefuroxime, gentamicin, nafcillin, piperacillin/tazobactam, tobramycin, and vancomycin. We considered as “nephrotoxic antibiotics use” at least one administration for at least one day of the abovementioned drugs.

*Site of infection*

*Site of infection* was defined as previously reported^1^ and categorized as pneumonia, intra-abdominal, urosepsis, skin and soft-tissue infections, central nervous system, endocarditis, catheter-related infection (e.g., indwelling catheters), unknown (e.g., for culture-negative sepsis, or the source was not identified), other (not specified above), and none (when the reason for the high lactate or hypotension was ultimately determined to not be from an infection).

**MISSING DATA**

*Urinary [TIMP-2]•[IGFBP7]*

The two biomarkers were measured after the end of the trial, randomly selecting 1000 urine samples for each timepoints (at enrollment and at 6 hours from enrollment) among the available and stored samples for the whole ProCESS trial (that consisted of 1341 patients). The samples were centrifuged right after collection, and the supernatant was frozen and stored at < -70°C. The supernatant was then thawed immediately prior to testing for two biomarkers of kidney stress, tissue inhibitor of metalloproteinases-2 (TIMP-2) and insulin-like growth factor binding protein 7 (IGFBP7) with the clinical immunoassay NephroCheck® Test (Astute Medical, Inc., San Diego, CA, USA) performed according to the manufacturer’s specifications.

It is possible that the random selection of the urine sample of some patients was not possible because their urine samples were not collected and/or unavailable for some of the following reasons:

- patients with septic shock could have been severely ill with oliguria or anuria, thus preventing the collection of urine samples. But only around 8% (12/153) of the patients missing [TIMP-2]•[IGFBP7] at enrollment and at 6 hours from enrollment were with AKI stage 3 using KDIGO criteria for urine output;
- patients with septic shock could have died in the first hours from enrollment, preventing the collection of urine samples. However, only 5% (7/153) of the patients with missing [TIMP-2]•[IGFBP7] at enrollment and at 6 hours from enrollment died in the same day of enrollment. Unfortunately, the exact hour of death was not available in hour dataset, and we cannot assess if death occurred within the first 6 hours;
- for the original purposes of the original ProCESS trial, collection of urine samples was not a mandatory condition, so it could be possible that other contingent situations could have prevented the site personnel from collecting the urine samples. Unfortunately, this condition was not reported in our dataset, and we cannot determine how many patients experienced that;
- the patient did not give the consent to perform tests on his/her biological samples or, after initial consent, he/she withdrew from the study preventing further collection/use of samples and/or data. Around 5% (7/153) of the patients missing [TIMP-2]•[IGFBP7] at 6 hours from enrollment underwent one of these conditions.

*Imputation of missing data*

The presence of missing data for [TIMP-2]•[IGFBP7] at enrollment or at 6 hours from enrollment, as well as for other variables (lactate, SOFA score, weight, and age) was addressed performing a multiple imputation by chained equation (MICE) in a separate sensitivity analysis reported extensively in this Supplemental Material. MICE was performed for missing data of the previously mentioned variables using predictive mean matching in about 25 datasets. e-Table 2 summarizes the variables with missing values pre-imputation while e-Table 3 summarizes the variables with missing values post-imputation.

**SENSITIVITY ANALYSIS**

**Methods**

We performed a sensitivity analysis after performing imputation for missing data (Supplemental Material – Imputation of Missing data) to address the possible selection bias related to excluding patients with missing values for urinary [TIMP-2]•[IGFBP7] both at 6 hours from enrollment and at enrollment. The sensitivity analysis cohort consisted of the patients from ProCESS trial after excluding only patients with end-stage renal disease (patients receiving chronic dialysis prior to a sepsis episode), reference sCr ≥4 mg/dL, or missing admission SCr (e-Figure 1), the same used for the ProGReSS-AKI study,^4^ the first ancillary study of the ProCESS trial.

The outcome *AKI* was defined as the presence of stage 2 or stage 3 with the addition of stage 1 with urinary [TIMP-2]•[IGFBP7] at 6 hours from enrollment >2.0 (ng/mL)^2^/1000. We selected the 6 hours timepoint since we and others previously reported that [TIMP-2]•[IGFBP7] is more informative when measured 6^10^ or 12 hours^11^ after initial therapy in patients with sepsis. The other outcomes were defined in the same way as for the primary analysis. The same statistical analysis described for the primary analysis was performed.

**Results**

*Baseline characteristics of the sensitivity analysis cohort and the phenotypes*

The secondary analysis cohort included 1243 patients (e-Figure 1). For 999 patients [TIMP-2]•[IGFBP7] at 6 hours from enrollment was available while for the remaining 244 patients the value was imputed as previously described. A total of 417 (33.5%) patients were in the α phenotype, 277 (22.3%) patients in β, 343 (27.6%) patients in γ, and 206 (16.6%) exhibited the δ phenotype. e-Table 4 describes the baseline characteristics of the secondary analysis cohort and the four phenotypes. Similar results to the primary analysis cohort were found regarding median age, race, and comorbidities distribution across phenotypes. Median age was higher in patients in the β and δ phenotypes compared to α and γ, Black race was more represented in the δ (31.1%) compared to α phenotype (19.9%, adjusted *P* for pairwise comparison <0.05). Most comorbidities have higher prevalence in patients in the β phenotype, in particular diabetes, arterial hypertension, congestive heart failure, cancer, prior myocardial infarction; while cerebral vascular disease, dementia and liver cirrhosis had higher prevalence in the δ phenotype. Consistent results with the primary analysis were found also regarding median Charlson Comorbidity Index, APACHE II score, SOFA score, lactate, reference sCr, and [TIMP-2]•[IGFBP7]. Similarly, the proportion of patients with [TIMP-2]•[IGFBP7] at 6 hours >2.0 (ng/mL)^2^/1000 was 19.5% in all the sensitivity analysis cohort, with the highest percentage reported again for δ phenotype (37.4%, adjusted *P<*0.05 for all the pairwise comparisons between δ and the other phenotypes).

*Outcomes in the sensitivity analysis cohort*

The results of the sensitivity analysis regarding the distribution of the outcomes across the four clinical phenotypes for sepsis were similar to what observed for the primary analysis in e-Table 5. Briefly, *AKI* was present in 632 (50.8%) patients; among them 377 (30.3%) had stage 2, 230 (18.5%) had stage 3, while 25 (2.0%) had stage 1B (biomarker positive stage 1).^12^ Of the remaining patients, 442 (35.6%) did not fulfill the KDIGO criteria for AKI within the first 24 hours after enrollment, while 169 (13.6%) patients had stage 1A (biomarker negative stage 1).^12^ β and δ phenotypes showed again the highest rate of *AKI* (70.8% and 79.6%, adjusted *P*>0.05 for their pairwise comparison between them), followed by γ phenotype (45.8%) and α with the lowest rate (27.6%). *AKD* was present in 22.7% of all the patients; the highest rates were observed for δ and β phenotypes (42.7% and 33.9% respectively). *CKD* was present in 35.8% of all the patients, but this percentage was statistically significantly higher (52.0%) in the β phenotype when compared to all the other phenotypes (adjusted *P*<0.05 for all pairwise comparisons). *AKI-on-CKD* was present in 18.6% of all the patients, but, slightly differently from the primary analysis, this percentage was higher for patients in the β and δ phenotype (37.5% and 27.7% respectively, adjusted *P*>0.05 for the pairwise comparison between β and δ phenotype). All-cause mortality at 90 days was 29.8%. Mortality varied across all the phenotypes (overall *P*<0.001) with the lowest rate in the α phenotype (13.2%) and the highest in the δ phenotype (47.6%). Additional details regarding the outcomes are provided in e-Table 5.

*Association between phenotypes and primary outcomes*

The logistic regression models showed that the phenotypes have different associations with *AKI* (in the overall cohort but also in patient with and without *CKD*) and with *AKD* (e-Table 6). Using the α phenotype as reference, also in the sensitivity analysis the strongest factor was always the δ-type, with adjusted odds ratios (ORs) of 11.53 (95% confidence interval (CI) 7.55-17.61, *P*<0.001) for the association with *AKI* in entire cohort, OR 9.79 (95%CI 5.84-16.41, *P*<0.001) for *AKI* in patients without *CKD*, OR 17.75 (95%CI 8.12-38.84, *P*<0.001) for *AKI* in patients with *CKD*, and 7.23 (95%CI 4.62-1131, *P*<0.001) for *AKD*. Patients in the β-type showed the second highest ORs. Patients in the γ-type had a significantly stronger association with the outcomes reported when compared to α. ORs for each phenotype are reported in e-Table 6 (right columns for the sensitivity analysis).

*Survival analysis and Cox proportional* *hazard* *models in the sensitivity analysis*

The results of the sensitivity analysis were consistent with the results observed in the primary analysis. Survival up to 90 days was different across all the four phenotypes (Log-rank test: *P*<0.001). The pairwise comparisons between phenotypes were all statistically significant (*P*<0.05) except the comparisons between β vs. γ phenotype (*P*=0.11). The lowest survival was shown for δ while the highest for α phenotype. Using α phenotype as reference, the adjusted HRs for death within 90 days were 2.36 (95%CI 1.69-3.30, *P*<0.001) for β, 2.45 (95%CI 1.77-3.39, *P*<0.001) for γ, and 4.04 (95%CI 2.88-5.68, *P*<0.001) for δ phenotypes (e-Figure 2A). The presence of *AKI* was associated with a lower survival (Log-rank test: *P*<0.001) with an adjusted HR of 1.99 (95%CI 1.59-2.49, *P*<0.001, e-Figure 2B). Moreover, *AKD* was associated as well with a lower survival (Log-rank test: *P*<0.001) with an adjusted HR of 3.13 (95% CI 2.51-3.90, *P*<0.001, e-Figure 2C). For *CKD*, the Log-rank test was significant (*P*<0.03), but the adjusted HR was not (1.11, 95% CI 0.88-1.41, *P*=0.39, e-Figure 2D). e-Table 7 showed different Cox proportional hazard models for different combination of the phenotypes and renal outcomes.

**SUPPLEMENTAL TABLES**

**e-Table 1**. Late AKI and renal replacement therapy by phenotype.

|  | **Total**  **(N. 1090)** | **α**  **(N. 364)** | **β**  **(N. 238)** | **γ**  **(N. 313)** | **δ**  **(N. 175)** |
| --- | --- | --- | --- | --- | --- |
| **Late AKI^a^** | 31 (2.8%) | 13 (3.6%) | 8 (3.5%) | 10 (3.2%) | 0 (0%) |
| **Renal replacement therapy^b^** | 34 (3.1%) | 3 (0.8%) | 13 (5.5%) | 6 (1.9%) | 12 (6.9%) |

^a^Acute kidney injury (AKI) occurring between 24-48h (second day) after enrollment, but not present at enrollment or within the first 24 hours (first day) according to KDIGO definition. This was different from our primary outcome that only included patients with AKI at enrollment or within the first 24 hours from enrollment.

^b^Renal replacement therapy within the first 48h

**e-Table 2.** Pre-imputation summary

| **VARIABLE** | **N. observations** | **Mean** | **Std. Dev.** | **Min** | **Max** |
| --- | --- | --- | --- | --- | --- |
| **[TIMP-2]•[IGFBP7] at enrollment,**  **(ng/mL)^2^/1000** | 929 | 1.87 | 2.61 | 0.00 | 10.00 |
| **[TIMP-2]•[IGFBP7] at 6 hours,**  **(ng/mL)^2^/1000** | 999 | 1.33 | 2.21 | 0.00 | 10.00 |
| **Lactate, mmol/L** | 1187 | 4.90 | 3.23 | 0.4 | 24 |
| **SOFA score** | 1237 | 7 | 4 | 0 | 19 |
| **Age, years** | 1242 | 61 | 16 | 18 | 90 |
| **Weight, Kg** | 1237 | 81.4 | 26.5 | 28.2 | 267.9 |

Abbreviations: IGFBP7 = insulin-like growth factor binding protein 7; SOFA = sequential organ failure assessment; TIMP-2 = tissue inhibitor metalloproteinases-2.

**e-Table 3.** Post-imputation summary

| **VARIABLE** | **N. observations** | **Mean** | **Std. Dev.** | **Min** | **Max** |
| --- | --- | --- | --- | --- | --- |
| **[TIMP-2]•[IGFBP7] at enrollment,**  **(ng/mL)^2^/1000** | 1243 | 1.82 | 2.46 | 0.00 | 10.00 |
| **[TIMP-2]•[IGFBP7] at 6 hours,**  **(ng/mL)^2^/1000** | 1243 | 1.31 | 2.12 | 0.00 | 10.00 |
| **Lactate, mmol/L** | 1243 | 4.87 | 3.17 | 0.4 | 24 |
| **SOFA score** | 1243 | 7 | 4 | 0 | 19 |
| **Age, years** | 1243 | 61 | 16 | 18 | 90 |
| **Weight, Kg** | 1243 | 81.4 | 26.4 | 28.2 | 267.9 |

Abbreviations: IGFBP7 = insulin-like growth factor binding protein 7; SOFA = sequential organ failure assessment; TIMP-2 = tissue inhibitor metalloproteinases-2.

**e-Table 4.** Baseline characteristics of the sensitivity analysis cohort and their distribution across the four clinical phenotypes for sepsis.

|  | **Total**  **(N. 1243)** | **α**  **(N. 417)** | **β**  **(N. 277)** | **γ**  **(N. 343)** | **δ**  **(N. 206)** |
| --- | --- | --- | --- | --- | --- |
| **Age, years** | 61 (50-74) | 57 (46-70) | 70 (57-79) | 60 (48-71) | 63 (55-76) |
| **Sex, male** | 688 (55.3%) | 221 (53.0%) | 162 (58.5%) | 182 (53.1%) | 123 (59.7%) |
| **Weight, kg** | 76 (64-92) | 77 (64-93) | 77 (66-92) | 75 (63-91) | 77 (64-93) |
| **Race ***  **White**  **Black**  **Other** | 870 (70.0%)  289 (23.3%)  84 (6.8%) | 302 (72.4%)  83 (19.9%)  32 (7.7%) | 202 (72.9%)  63 (22.7%)  12 (4.3%) | 235 (68.5%)  79 (23.0%)  29 (8.5%) | 131 (63.6%)  64 (31.1%)  11 (5.3%) |
| **Diabetes** | 406 (32.7%) | 102 (24.5%) | 121 (43.7%) | 117 (34.1%) | 66 (32%) |
| **Cardiovascular disease**** | 806 (64.8%) | 243 (58.3%) | 222 (80.1%) | 219 (63.8%) | 122 (59.2%) |
| Arterial hypertension | 719 (57.8%) | 218 (52.3%) | 200 (72.2%) | 188 (54.8%) | 113 (54.9%) |
| Congestive heart failure | 140 (11.3%) | 35 (8.4%) | 56 (20.2%) | 29 (8.5%) | 20 (9.7%) |
| Prior myocardial infarction | 131 (10.5%) | 33 (7.9%) | 44 (15.9%) | 32 (9.3%) | 22 (10.7%) |
| Cerebral vascular disease | 119 (9.6%) | 25 (6.0%) | 34 (12.3%) | 27 (7.9%) | 33 (16.0%) |
| Peripheral vascular disease | 92 (7.4%) | 23 (5.5%) | 27 (9.7%) | 22 (6.4%) | 20 (9.7%) |
| Chronic respiratory disease | 281 (22.6%) | 87 (20.9%) | 76 (27.4%) | 78 (22.7%) | 40 (19.4%) |
| **Active cancer** | 227 (18.3%) | 58 (13.9%) | 80 (28.9%) | 63 (18.4%) | 26 (12.6%) |
| **Dementia** | 98 (7.9%) | 26 (6.2%) | 22 (7.9%) | 19 (5.5%) | 31 (15.0%) |
| **Liver cirrhosis** | 79 (6.4%) | 9 (2.2%) | 14 (5.1%) | 28 (8.2%) | 28 (13.6%) |
| **Ulcer disease** | 68 (5.5%) | 28 (6.7%) | 19 (6.9%) | 9 (2.6%) | 12 (5.8%) |
| **HIV infection** | 34 (2.7%) | 8 (1.9%) | 9 (3.2%) | 12 (3.5%) | 5 (2.4%) |
| **Charlson Comorbidity Index** | 2 (1-4) | 1 (0-2) | 3 (2-6) | 2 (1-3) | 2 (1-4) |
| **APACHE II score** | 19 (15-25) | 16 (12-20) | 21 (18-26) | 19 (15-23) | 25 (20-32) |
| **SOFA score** | 7 (4-9) | 5 (3-7) | 7 (5-9) | 6 (4-9) | 10 (8-12) |
| **Lactate, mmol/L** | 4.4 (2.6-6.0) | 4.3 (2.2-5.3) | 3.8 (2.0-5.0) | 4.3 (2.6-5.9) | 6.8 (4.9-9.4) |
| **Reference creatinine** | 1.0 (0.8-1.3) | 1.0 (0.8-1.2) | 1.1 (0.9-1.4) | 1.0 (0.8-1.2) | 1.0 (0.8-1.2) |
| **[TIMP-2]•[IGFBP7] > 2.0 (ng/mL)^2^/1000 ***** | 242 (19.5%) | 52 (12.5%) | 45 (16.2%) | 68 (19.8%) | 77 (37.4%) |
| **[TIMP-2]•[IGFBP7],**  **(ng/mL)^2^/1000 ***** | 0.38  (0.15-1.39) | 0.25  (0.11-0.64) | 0.53  (0.18-1.31) | 0.36  (0.15-1.53) | 1.00  (0.34-3.09) |

Categorical variables are presented as numbers (%), continuous variables as medians (interquartile range).

*White race corresponds to white/Caucasian; Black race corresponds to black/African American; Other race corresponds to Asian, American Indian or native Alaskan, Native Hawaiian or other Pacific islander, unknown, or other.

**Presence of any among arterial hypertension, congestive heart failure, previous myocardial infarction, cerebral vascular disease, and peripheral vascular disease.

***[TIMP-2]•[IGFBP7] at 6 hours from enrollment. The value was imputed for 244 patients.

Abbreviations: APACHE = acute physiology and chronic health evaluation; IGFBP7 = insulin-like growth factor binding protein 7; SOFA = sequential organ failure assessment; TIMP-2 = tissue inhibitor metalloproteinases-2.

**e-Table 5.** Distribution of the outcomes across the four clinical phenotypes for sepsis in the sensitivity analysis cohort.

|  | **Total**  **(N. 1243)** | **α**  **(N. 417)** | **β**  **(N. 277)** | **γ**  **(N. 343)** | **δ**  **(N. 206)** | ***P*** |
| --- | --- | --- | --- | --- | --- | --- |
| **PRIMARY OUTCOME** | | | | | | |
| **AKI** | 632 (50.8%) | 115 (27.6%)^a^ | 196 (70.8%)^b^ | 157 (45.8%)^c^ | 164 (79.6%)^b^ | **<0.001** |
| **SECONDARY OUTCOMES** | | | | | | |
| **AKD** | 282 (22.7%) | 39 (9.4%)^a^ | 94 (33.9%)^b^ | 61 (17.8%)^c^ | 88 (42.7%)^b^ | **<0.001** |
| **CKD** | 445 (35.8%) | 131 (31.4%)^a^ | 144 (52%)^b^ | 98 (28.6%)^a^ | 72 (35%)^a^ | **<0.001** |
| **AKI-on-CKD**  AKI - / CKD -  AKI - / CKD +  AKI +/ CKD - | 231 (18.6%)  397 (31.9%)  214 (17.2%)  401 (32.3%) | 28 (6.7%)^a^  199 (47.7%)^a^  103 (24.7%)^a^  87 (20.9%)^a^ | 104 (37.5%)^b^  41 (14.8%)^b^  40 (14.4%)^b,c^  92 (33.2%)^b^ | 42 (12.2%)^a^  130 (37.9%)^c^  56 (16.3%)^b^  115 (33.5%)^b^ | 57 (27.7%)^b^  27 (13.1%)^b^  15 (7.3%)^c^  107 (51.9%)^c^ | **<0.001** |
| **Mortality at 90 days** | 371 (29.8%) | 55 (13.2%)^a^ | 106 (38.3%)^b,c^ | 112 (32.7%)^b^ | 98 (47.6%)^c^ | **<0.001** |

Categorical variables are presented as numbers (%). If overall *P*<0.05, pairwise comparisons between phenotypes was performed using Bonferroni correction. Values in the same row not sharing the same superscript letter are significantly different at adjusted *P*<0.05.

Abbreviations: AKD = acute kidney disease; AKI = acute kidney injury; CKD = chronic kidney disease.

**e-Table 6.** Logistic regression models showing the association of the clinical phenotypes for sepsis with AKI (evaluated in the whole cohort, in patients without CKD and in patient with CKD) and AKD in the primary and sensitivity analysis cohort.

| **Models** | **Primary analysis** | | **Sensitivity Analysis** | |
| --- | --- | --- | --- | --- |
| **Dependent variable:**  ***AKI* (whole cohort)** | **Adjusted^a^ OR (95% CI)** | ***P*** | **Adjusted^a^ OR (95% CI)** | ***P*** |
| Number of patients | 1090 |  | 1243 |  |
| Phenotype β (ref. α) | 8.32 (5.55 - 12.48) | <0.001 | 7.40 (5.10 - 10.74) | <0.001 |
| Phenotype γ (ref. α) | 2.84 (2.01 - 3.99) | <0.001 | 2.45 (1.79 - 3.37) | <0.001 |
| Phenotype δ (ref. α) | 12.33 (7.81 - 19.47) | <0.001 | 11.53 (7.55 - 17.61) | <0.001 |
| **Dependent variable:**  ***AKI* in patients without *CKD*** | **Adjusted^a^ OR (95% CI)** | ***P*** | **Adjusted^a^ OR (95% CI)** | ***P*** |
| Number of patients | 703 |  | 798 |  |
| Phenotype β (ref. α) | 6.20 (3.66 - 10.51) | <0.001 | 5.15 (3.20 - 8.30) | <0.001 |
| Phenotype γ (ref. α) | 2.70 (1.79 - 4.05) | <0.001 | 2.16 (1.49 - 3.14) | <0.001 |
| Phenotype δ (ref. α) | 11.93 (6.82 - 20.87) | <0.001 | 9.79 (5.84 - 16.41) | <0.001 |
| **Dependent variable:**  ***AKI* in patients with *CKD*** | **Adjusted^a^ OR (95% CI)** | ***P*** | **Adjusted^a^ OR (95% CI)** | ***P*** |
| Number of patients | 387 |  | 445 |  |
| Phenotype β (ref. α) | 12.38 (6.19 - 24.79) | <0.001 | 14.83 (7.55 - 29.12) | <0.001 |
| Phenotype γ (ref. α) | 3.16 (1.65 - 6.04) | 0.001 | 3.37 (1.80 - 6.28) | <0.001 |
| Phenotype δ (ref. α) | 12.91 (5.67 - 29.36) | <0.001 | 17.75 (8.12 - 38.84) | <0.001 |
| **Dependent variable:**  ***AKD*** | **Adjusted^a^ OR (95% CI)** | ***P*** | **Adjusted^a^ OR (95% CI)** | ***P*** |
| Number of patients | 1090 |  | 1243 |  |
| Phenotype β (ref. α) | 7.63 (4.55 - 12.80) | <0.001 | 5.31 (3.41 - 8.28) | <0.001 |
| Phenotype γ (ref. α) | 2.98 (1.79 - 4.97) | <0.001 | 2.24 (1.44 - 3.48) | <0.001 |
| Phenotype δ (ref. α) | 9.18 (5.44 - 15.51) | <0.001 | 7.23 (4.62 - 11.31) | <0.001 |

^a^OR were adjusted for: age, sex, weight (after logarithm transformation), race, diabetes, arterial hypertension, congestive heart failure, chronic respiratory disease, cancer.

Abbreviations: AKI = acute kidney injury; AUC = area under curve; CI = confidence interval; CKD = chronic kidney disease; OR = odds ratio;

**e-Table 7**. Cox proportional hazard models for death within 90 days with different combinations of the phenotypes and renal outcomes in the sensitivity analysis cohort.

|  | **Model A** | | **Model B** | | **Model C** | |
| --- | --- | --- | --- | --- | --- | --- |
|  | **HR (95% CI) ^a^** | ***P*** | **HR (95% CI) ^a^** | ***P*** | **HR (95% CI) ^a^** | ***P*** |
| **Phenotype β (ref. α)** | 1.99 (1.41 - 2.82) | <0.001 | 1.86 (1.32 - 2.62) | 0.004 | 2.34 (1.66 - 3.28) | <0.001 |
| **Phenotype γ (ref. α)** | 2.24 (1.62 - 3.12) | <0.001 | 2.30 (1.66 - 3.19) | <0.001 | 2.45 (1.77 - 3.39) | <0.001 |
| **Phenotype δ (ref. α)** | 3.30 (2.31 - 4.71) | <0.001 | 3.04 (2.14 - 4.31) | <0.001 | 4.03 (2.87 - 5.66) | <0.001 |
| **AKI** | 1.54 (1.22 - 1.94) | <0.001 | / | / | / | / |
| **AKD** | / | / | 2.69 (2.14 - 3.38) | <0.001 | / | / |
| **CKD** | / | / | / | / | 1.06 (0.83 - 1.35) | 0.67 |

^a^Adjusted for age, sex, weight (after logarithm transformation), race, diabetes, and comorbidities with significant *P*<0.10 at univariate analysis for mortality at 90 days (not shown). The comorbidities entered in all above models were: cardiovascular disease (presence of any between arterial hypertension, history of congestive heart failure, prior myocardial infarction, cerebral vascular disease, or peripheral vascular disease), cancer, dementia, liver cirrhosis, and HIV infection.

Abbreviations: AKI = acute kidney injury; CI = confidence interval; CKD = chronic kidney disease; HIV = human immunodeficiency virus; HR = hazard ratio.

**SUPPLEMENTAL FIGURES**

**e-Figure 1:** Study Flow.

The figure shows how we obtained our primary and sensitivity analysis cohort.

ProCESS^1^ was a multicenter, randomized clinical trial of three different resuscitation strategies in patients with septic shock that enrolled 1341 patients without finding any difference in mortality and need of organ support between the different strategies. Kellum and colleagues in a follow-up study^2^ specifically evaluated if the presence and the severity of acute kidney injury (AKI) were affected by the different resuscitation strategies. No association was found between different resuscitation strategies and development of AKI and its severity in a subgroup of 1243 patients resulted after excluding patients for whom the assessment of AKI was impossible or unreliable, such as patients with end-stage kidney disease (chronic dialysis not related to the current sepsis episode), reference serum creatinine ≥4 mg/dL, or missing enrollment serum creatinine. In our study, from the cohort of 1243 patients, we excluded patients with missing the measurements of urinary [TIMP-2]•[IGFBP7] at 6 hours from enrollment and at enrollment.


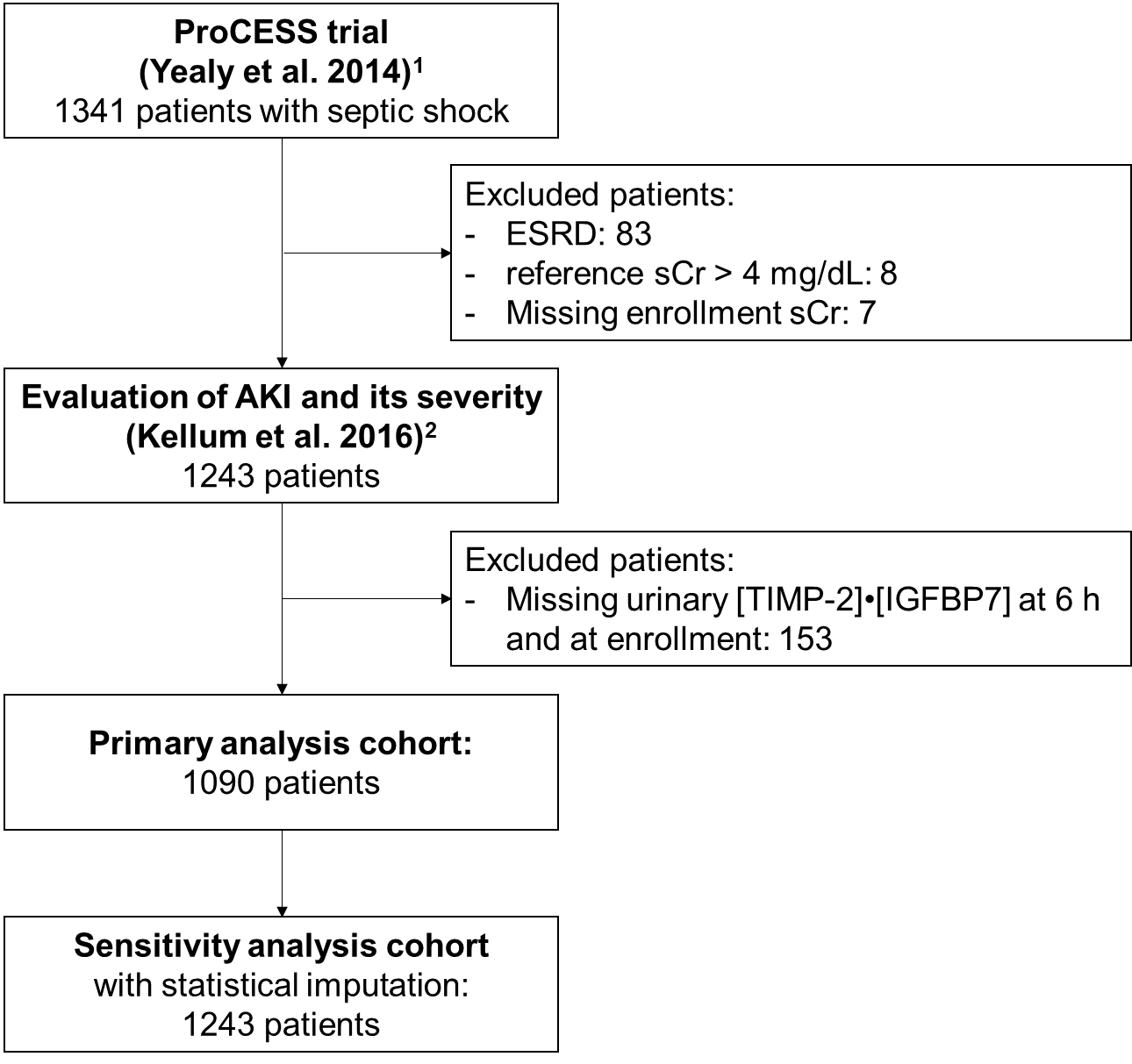


^1^Yealy DM, Kellum JA, Huang DT, et al. A randomized trial of protocol-based care for early septic shock. N Engl J Med. 2014;370(18):1683-1693.

^2^Kellum JA, Chawla LS, Keener C, et al. The Effects of Alternative Resuscitation Strategies on Acute Kidney Injury in Patients with Septic Shock. Am J Respir Crit Care Med. 2016;193(3):281-287.

Abbreviations: ESRD = end-stage renal disease; IGFBP7 = insulin-like growth factor binding protein 7; sCr = serum creatinine; TIMP-2 = tissue inhibitor of metalloproteinases-2.

**e-Figure 2:** Covariate-adjusted survival curves at 90 days obtained from Cox proportional hazard models in the sensitivity analysis.

**
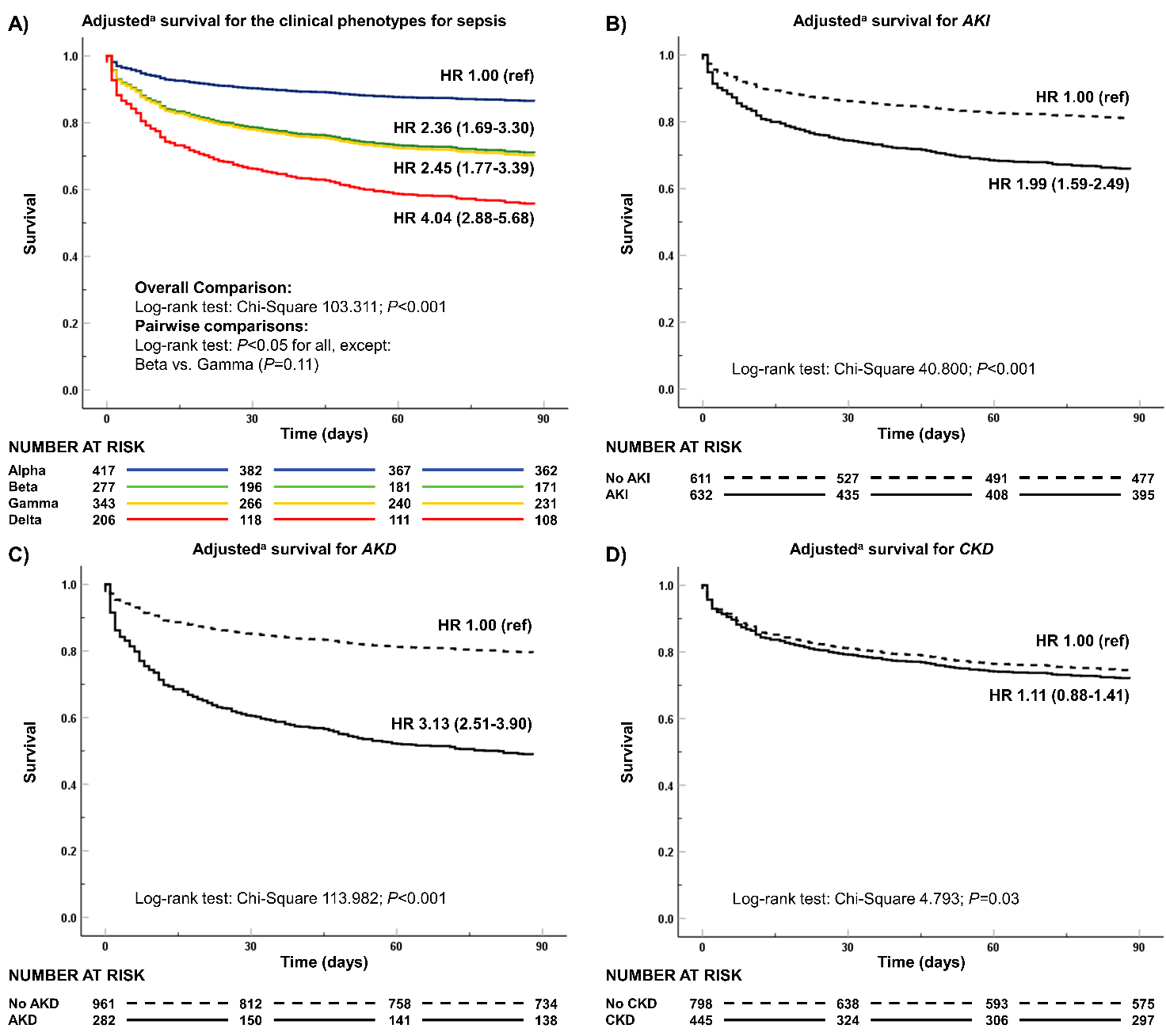
**

^a^Hazard Ratios (95% confidence interval) and survival curves in each panel were obtained from different Cox proportional hazard models. In each model, the variable shown in the figure was evaluated separately only with the other adjusting covariates. The adjusting covariates were: age, sex, weight (after logarithm transformation), race, diabetes, and comorbidities with significant *P*<0.10 at univariate analysis for mortality at 90 days (not shown). The comorbidities entered in all the above models were: cardiovascular disease (presence of any between arterial hypertension, history of congestive heart failure, prior myocardial infarction, cerebral vascular disease, or peripheral vascular disease), cancer, dementia, liver cirrhosis, and HIV infection.

**Abbreviations**: AKI = acute kidney injury; CI = confidence interval; CKD = chronic kidney disease; HIV = human immunodeficiency virus; HR = adjusted hazard ratio.

**PROPORTIONAL HAZARDS ASSUMPTION TESTING FOR COX MODELS**

In the primary analysis cohort, we ran Cox proportional hazard models with all-cause mortality, censored at 90-days, as the outcome and separately for the phenotypes, AKI, AKD, and CKD as a stratum. Then we drew the figures (e-Figure 3) showing the log minus log function for cumulative survival for the sepsis phenotypes, *AKI*, *AKD* and *CKD* (3-figure 3A, 3B, 3C and 3D respectively).

The parallels curves in the four plots confirm the respect of the proportional hazards assumption.

**Abbreviations**: AKI = acute kidney injury; AKD = acute kidney disease; CKD = chronic kidney disease.

**e-Figure 3A**


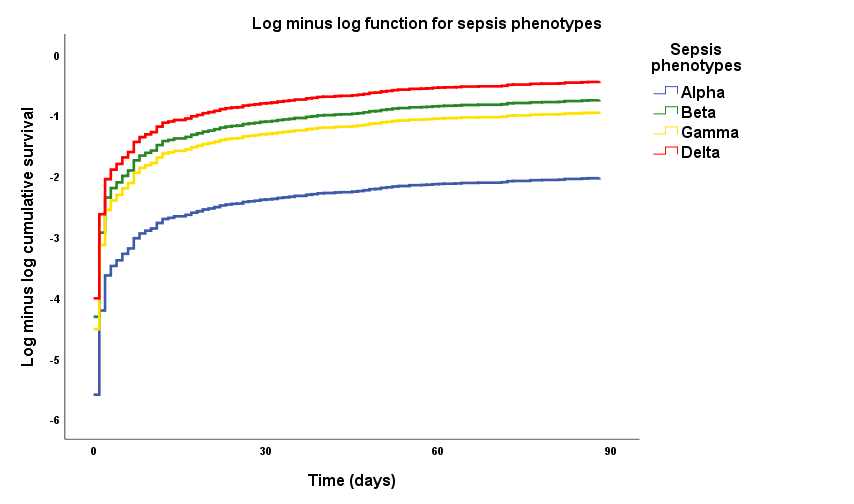


**e-Figure 3B**


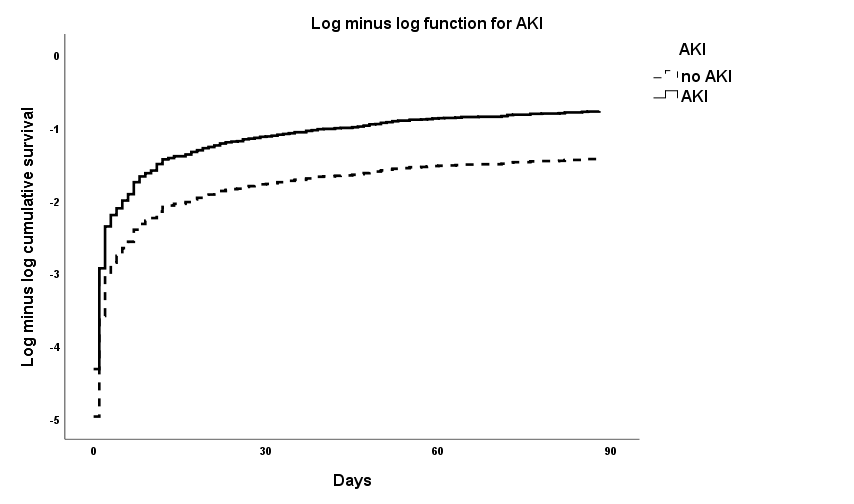


**e-Figure 3C**


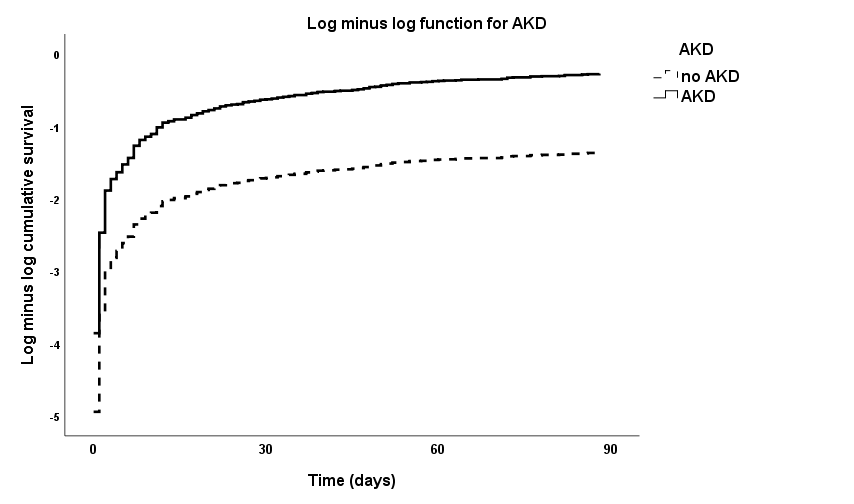


**e-Figure 3D**


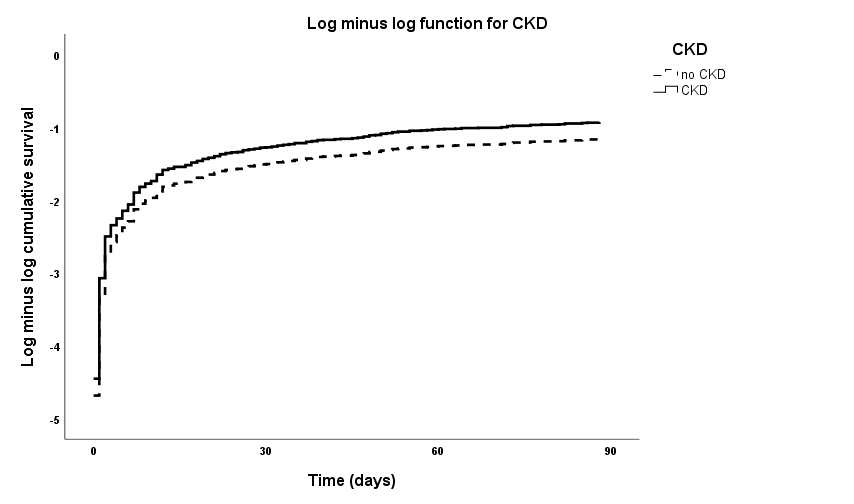


**SUPPLEMENTAL** **MATERIAL** **REFERENCES**

1. Yealy DM, Kellum JA, Huang DT, et al. A randomized trial of protocol-based care for early septic shock. *N Engl J Med.* 2014;370(18):1683-1693.

2. Dellinger RP, Levy MM, Carlet JM, et al. Surviving Sepsis Campaign: international guidelines for management of severe sepsis and septic shock: 2008. *Crit Care Med.* 2008;36(1):296-327.

3. Bone RC, Balk RA, Cerra FB, et al. Definitions for sepsis and organ failure and guidelines for the use of innovative therapies in sepsis. The ACCP/SCCM Consensus Conference Committee. American College of Chest Physicians/Society of Critical Care Medicine. *Chest.* 1992;101(6):1644-1655.

4. Kellum JA, Chawla LS, Keener C, et al. The Effects of Alternative Resuscitation Strategies on Acute Kidney Injury in Patients with Septic Shock. *Am J Respir Crit Care Med.* 2016;193(3):281-287.

5. Kidney Disease: Improving Global Outcomes (KDIGO) Acute Kidney Injury Work Group. KDIGO Clinical Practice Guideline for Acute Kidney Injury. *Kidney Int Suppl.* 2012;2(1):1-138.

6. Levey AS, Bosch JP, Lewis JB, Greene T, Rogers N, Roth D. A more accurate method to estimate glomerular filtration rate from serum creatinine: a new prediction equation. Modification of Diet in Renal Disease Study Group. *Ann Intern Med.* 1999;130(6):461-470.

7. Seymour CW, Kennedy JN, Wang S, et al. Derivation, Validation, and Potential Treatment Implications of Novel Clinical Phenotypes for Sepsis. *JAMA.* 2019;321(20):2003-2017.

8. Opal SM, Laterre PF, Francois B, et al. Effect of eritoran, an antagonist of MD2-TLR4, on mortality in patients with severe sepsis: the ACCESS randomized trial. *JAMA.* 2013;309(11):1154-1162.

9. Bernard GR, Vincent JL, Laterre PF, et al. Efficacy and safety of recombinant human activated protein C for severe sepsis. *N Engl J Med.* 2001;344(10):699-709.

10. Fiorentino M, Xu Z, Smith A, et al. Serial Measurement of Cell-cycle Arrest Biomarkers [TIMP-2]•[IGFBP7] and Risk for Progression to Death, Dialysis or Severe Acute Kidney Injury in Patients with Septic Shock. *Am J Respir Crit Care Med.* 2020.

11. Nusshag C, Rupp C, Schmitt F, et al. Cell Cycle Biomarkers and Soluble Urokinase-Type Plasminogen Activator Receptor for the Prediction of Sepsis-Induced Acute Kidney Injury Requiring Renal Replacement Therapy: A Prospective, Exploratory Study. *Crit Care Med.* 2019;47(12):e999-e1007.

12. Ostermann M, Zarbock A, Goldstein S, et al. Recommendations on Acute Kidney Injury Biomarkers From the Acute Disease Quality Initiative Consensus Conference: A Consensus Statement. *JAMA Netw Open.* 2020;3(10):e2019209.
